# The Outcome of a Self-Efficacy Enhancement Program for Cervical Cancer Screening among Women in Phrasaeng District, Suratthani Province, Thailand

**DOI:** 10.31557/APJCP.2020.21.7.2075

**Published:** 2020-07

**Authors:** Ornuma Bunkarn, Kiatkamjorn Kusol, Thidarat Eksirinimit

**Affiliations:** *School of Nursing, Walailak University, Thasala, Nakhon Si Thammarat, Thailand.*

**Keywords:** Self-efficacy enhancement program, cervical cancer screening, women

## Abstract

**Objective:**

The purpose of this research was to examine the effect of a self-efficacy enhancement program on perceived self-efficacy and cervical cancer screening among women.

**Methods:**

This research was quasi-experimental research, using the subjects consisted of 130 samples; each of the experimental and control group consisted of 65 samples. The experimental group received a self-efficacy enhancement program. The control group received knowledge and usual care by health personnel in the health promoting hospital. The duration of the research was 8 weeks. The self-efficacy was testing the reliability equal to 0.90. Data were analyzed using descriptive statistics, Chi-square, and t-test.

**Results:**

The mean scores of perceived self-efficacy between the experimental and control group before using the program (M=2.18, S.D.=0.40; M=2.22, S.D.=0.39), there was no significantly different (p> 0.05). The mean scores of perceived self-efficacy between the experimental and control group after using the program (M=4.11, S.D.=0.44; M=2.28, S.D.=0.42), there was a significantly different (p< 0.001). The mean scores of perceived self-efficacy of the experimental group before and after using the program were higher, (M=2.18, S.D.=0.40; M=4.11, S.D.=0.44), there was a significantly different (p< 0.001). The experimental group came to screening 64 people (98.5 %), and the control group came to screening eight people (12.3 %), calculated ratio as 8:1 and compared the cervical cancer screening in the experimental and control group had differences significantly (p< 0.001).

**Conclusion:**

The self-efficacy enhancement program, resulting in increased screening rates and screened after the first week by having most screening tests in the community. Therefore, the program should be applied, and proactive services should be provided for women to have access in community and a health service place.

## Introduction

Cervical cancer was a major public health problem worldwide. In women, new cases found more than 500,000 per year, and deaths 250,000 per year, more common in developing countries (WHO, 2018). Thailand had the most common cervical cancer, second only to breast cancer; each year, there were new patients an additional 10,000 per year and deaths approximately 5,000 per year (Ministry of Public Health, Strategy and Planning Division, 2017).

Cervical cancer caused human papillomavirus, which transmitted through sexual contact (Sidabutar et al., 2018). The period of infection before cervical cancer was about 10-20 years (Ministry of Public Health, Department of Health, 2017). There was HPV infection in the epithelium at the transformation zone of the cervix of progressive, resulting in abnormal cells and epithelium to become carcinoma, for the additional factors caused by first sexual intercourse younger than 16 years, and more than 10 pregnancies (Kelly et al., 2017). Most women have symptoms that appear late in the disease and have spread to various organs, for common physical symptoms include; abdominal pain, vaginal bleeding, pale, thin, and tired conditions (Maniar and Wei, 2017).

The Ministry of Public Health has a policy to campaign for target women aged 30-60 years who came screening for cervical cancer to the target group by 80 percent, which to reduce the illness with cervical cancer rate (Ministry of Public Health, Department of Medical Services, Cancer Institute, 2018). The national survey found that 10.4 million women were screened for cervical cancer for 5.4 million, accounting for 51.58 percent, which was still lower than the criteria set by Public Health (Ministry of Public Health, 2019). Among 200,000 women in the target group determined by Suratthani Provincial Public Health Office 55,000 of whom received screening, accounting for 27.17 percent, which was a resulted in that had not met the target and less than at the national level (Suratthani Provincial Public Health Office, 2018).

The study of the literature review found that women lack knowledge in cervical cancer, being abashed of official, having either no time, afraid to the pain of screening, fearful of finding abnormally selection and incorrect attitudes toward the screening process (Gatumo et al., 2019; Rimande-Joel and Ekenedo, 2019). Including lack of awareness and not the importance of cervical cancer screening (Chosamata et al., 2015). Besides, some women were not aware of the importance of screening, not receiving news and information from health personnel, preventing women from learning for self-efficacy development, which can help them decide on for cervical cancer screening (Alnafisah et al., 2019). Therefore, proactive work should be of the providing of cervical cancer screening services in the community encourages women to access the screening because it helps women to have better expectations for themselves and their enthusiasm (Sakaew et al., 2017). The strengthening develops of the modeling of women, which to be able to educate women correctly, and together with enhancing the self-efficacy of women concurrently, it builds confidence in the capacity of women to make their own decisions (Suksawat et al., 2017).

Therefore, the researcher applied four strategies based on the self-efficacy concept proposed by Bandura (Bandura, 1997). Including; 1) Mastery experience was focus on issues about cervical cancer screening and successful exchange of experiences, including was important for women to learn by using the analytical process for cervical cancer screening and creating a learning experience on an important issue for women (Kim et al., 2018). 2) Modeling communicated through direct experience and through the media, which women to create motivation processes emulating successful others (Izadirad et al., 2017). 3) Emotional arousal was helping women aware realize the importance of cervical cancer screening (Chosamata et al., 2015). And 4) Verbal persuasion was to encourage and appreciate women, resulting in women recognizing the importance and motivation for cervical cancer screening (Izadirad et al., 2017). Therefore, the program has organized the activities for women inexperienced directly experience in learning about cervical cancer. Learning through the models that have successful experience for cervical cancer screening, including being persuaded, motivated, and encourage continuous emotions, to make women knew, understanding cervical cancer, and aware of the importance of screening. The integration of all 4 strategies will help women become more confident in their performance until the expectations that can decide for cervical cancer screening. Therefore, the researchers believed that the development of this program using the self-efficacy theory would help women to have higher performance. 

The assessment of perceived self-efficacy included 3 dimensions (Bandura, 1977). Such as 1) Strength dimension was an assessment of actions that make women confident. 2) The generality dimension emphasized self-assessment to build confidence. And 3) Magnitude dimension aiming to have self-expectations that can lead to success. Women had a high level in all 3 dimensions as a result of women having the determination to succeed (Sidabutar et al., 2018). 

For reducing the mentioned problems, the researchers have realized the importance of promoting their competencies to make women screened for cervical cancer according to the criteria set by the Ministry of Public Health.Therefore, it was necessary to develop a self-efficacy enhancement program for cervical cancer screening among women, to be used as for secondary cervical cancer prevention and develop cervical cancer screening services, focusing on proactive work and participation among health personnel, modeling and health volunteers in encouraging women to have the confidence to decide for cervical cancer screening by themselves. Resulting in cervical cancer screening rates among women were reduced the incidence and death rate of cervical cancer.

## Materials and Methods

This research was quasi-experimental research to develop a program to promote the self-efficacy of women towards cervical cancer screening.

*Population and sample*

The population was women aged 30- 60 years with no prior experience of cervical cancer screening in Phrasaeng District, Suratthani, Thailand. 

The sample size was calculated by G*power program 3.1.2 (Hair et al., 2010). The referring to the research of Maneechot et al. (2011), with a sample size of 55 people, the researcher has set the sample size to prevent the sample group from dropping during the research study, 18 % added 10 people as sample 65 people per group. 

*Random sample*

The researcher analyzed with the SPSS program version 22. Statistic employed included;

1. The researcher randomized the samples from the women aged 30-60 years in 7 sub-districts, Phrasaeng District, Suratthani Province. 

2. Stratified random sampling by drawing the names from 7 sub-districts to 2 sub-districts. The samples were obtained from Trikhung and Saku Sub-district, both of which have a similar context. The researcher examined qualifications women following the selection criteria for each sub-district. After that, the villages randomly selected from 2 sub-districts. 

3. The randomized sampling using from the HDC online registry. The randomly selected number of women were from each village into the experimental and control group, with 65 people per group.

*Research instruments*

The research instruments were divided into 2 parts as follows: 1) The instrument used to the experiment, including a self-efficacy program for cervical cancer screening, nursing manuals, and women manuals, 2) Data collection instruments were general information and perceived self-efficacy questionnaires.

The self-efficacy enhancement program; the researcher checked the content in the program from 5 experts to have more clear content.

*Data collection methods*

The duration of the research was 8 weeks, which consisted of 2 weeks in preparation period, 2 weeks for program implementation and evaluation of perceived self-efficacy after using the program for 4 weeks, follow up for screening for cervical cancer, which the details were as follows;

1. Preparation stage: Before the data collection process, the researcher had engaged in the two-week preparation process as detailed below;

Week 1: The researcher met with the volunteers and women models to inform them of the study’s objectives, methodologies, benefits, and cooperation in conducting the study. The researcher prepared women models using questions and answers in sharing the first experience with women in the experimental group. The researcher also asked the volunteers to assist in notifying women recruited through the sampling method to attend the study activities as scheduled. 

 Week 2: The experimental group and the control group 

- The researcher organized a meeting with the participant women at the village meeting hall to provide in-depth explanations concerning the study’s objectives, actions, and woman’s rights. 

- The researcher retrieved the completed consent forms from the control group participants and also asked for their cooperation in completing the general information questionnaires and perceived self-efficacy questionnaires before distributing the manual for women. 

*2. Experiment stage*

*2.1 The experimental group was required to attend all of the program activities which scheduled as follows*; 

Week 3: Emphasizing the use of mastery experiences and modeling in carrying out the activities. The researcher introduced the participants to the knowledge of cervical cancer and also presented cervical cancer screening equipment and methods, learning models with successful female, and sharing experiences. In group activities, the participants split into groups of eight, and each participant took a turn sharing experiences within the groups. Then, representatives were randomly called upon from four groups for class sharing. 

Week 4: Emphasizing the use of verbal persuasion and emotional arousal in carrying out activities. The researcher provided guidelines for verbal persuasion by practical training in which the participants worked in pairs to verbally persuading friends about receiving screening tests. 

*2.2 The control group*

Week 3: The research control group organized activities with health personnel to provide knowledge about cervical cancer and recommendations regarding screening as per the regular general education.

Week 4: The research control group read the handbook for women at home.

3. Evaluation Stage: Both in the experimental and control groups

Week 5-8: The experimental group and the control group screened at Health Promoting Hospital every day on official days. The researcher and teams provided screening services, and recorded the screening data for both the experimental and control groups. 

*Statistical Analysis*

The researcher analyzed data with the SPSS program version 22. The statistic employed included; 

1. Descriptive Statistics was used to analyze the demographic data such as frequencies, percentage, mean and standard deviation.

2.Before the experiment, test similarity of the demographic data between the experimental and control groups. Chi-square, Fisher’s exact test were used for nominal data, and independent t-test was used for interval scale data. Results suggested a substantial similarity. 

3. Comparing mean scores of self-efficacy between the experimental and control groups by employing independent t-test, and comparing the number of screening to determine differences between the experimental and control groups using Chi-square test. 

*Ethical Approval*

This research has been officially approved by the Office of Human Research Ethics Committee of Walailak University as affirmed by the approval letter numbered WU-19-125-01, 24^th^ in July 2019.

## Results

*Sample general data*

For the sample general data, 36.9 percent of the participants were within a 30- 40 age range. A majority of 79.3 percent were married, 96.2 percent most of them have Buddhists. 71.5 percent have primary education, 34.2 percent received news about cervical cancer from health volunteers, 28.8 percent received from health care teams. Most of them did not have a medical history of Vaginitis, Leukorrhea, or post intercourse bleeding a large percentage of 83.8. The reasons did not screen as a lack of time 29.8 percent, followed by shying health care providers 28.0 percent. Statistics demonstrated, indicating no significant difference (p > 0.05) between the experimental and control groups (Table 1).

*The outcome of applying the self-efficacy program to the sample group*

A comparison of pre-program self-efficacy mean scores of women aged 30- 60 of both the experimental and control groups were at a low level in all 3 aspects. A comparison of pre-program self-efficacy mean scores in each dimension between the experimental and the control groups suggested no statistically significant difference (p> 0.05), as illustrated in Table 2.

A comparison of post-program self-efficacy mean scores of women aged 30- 60 between the experimental and control groups showed that the experimental group exhibited an increase of 4.11 from 2.18. A comparison of post-program self-efficacy mean scores in each dimension between the experimental and the control groups suggested a significant difference (p < 0.001), as illustrated in Table 3.

The number of cervical cancer screening tests was in the experimental group after using the self-efficacy enhancement program completed for 4 weeks. After that, in 1st week, women were for cervical cancer screening 61 participants (93.9%). In the 2^nd^ week, women were screening 3 participants (4.6%). The control group came to receive the screening 8 participants (12.3%) in the 1st week, showed that after receiving the self-efficacy enhancement program, make women more confident for cervical cancer screening. 

The results of comparing the screening rates between the experimental group and the control group; the amount was 64: 8 or 8: 1, after receiving the program marked women came to for cervical cancer screening. Most of the experimental group and the control group came to cervical cancer screening in the first weeks after applying for the program entirely.

The effectiveness calculation after using the self-efficacy program compared with the criteria, the number of cervical cancer screening among women according to the requirements of the Ministry of Public Health. There were 80% 5-year cumulative targets set. Therefore, the effectiveness of cervical cancer screening of women in the experimental group was equal to 123.13 percent, and the effectiveness of cervical cancer screening of women in the control group was similar to 15.38 percent. The women received the program increased their self-efficacy due to the development of their self-efficacy and cognitive processes for self-motivation. There was positive thinking that can decide for cervical cancer screening (Table 4).

A comparison of cervical screening rates of the sample groups after the self-efficacy enhancement program showed that after the program, 64 participants (98.5%) in the experimental group came to receive the screening test while the control group was 8 participants (12.3%). The experimental group came to cervical cancer screening, after receiving the self-efficacy enhancement program for 98.5 percent, which was more than the criteria of the Ministry of Public Health for 18.5 percent. Statistics demonstrated, indicating significant difference (p< 0.001) between the experimental and control groups (Table 5).

**Table 1:** General Data of the Study Participants (n= 130)

Variables	Total	Control	Experiment	*P*-value
	(n = 130)	(n = 65)	(n = 65)	
	Number (%)	Number (%)	Number (%)	
(Min = 30, Max = 60, Mean = 44.51, SD = 8.27) Age				
30 – 40 year	48 (36.9)	25 (38.5)	23 (35.4)	0.91
41 – 50 year	45 (34.6)	21 (32.3)	24 (36.9)	
51 – 60 year	37 (28.5)	19 (29.2)	18 (27.7)	
Marital status				
Married	103 (79.3)	54 (83.0)	49 (75.3)	0.59
Single/Divorced/Separated	27 (20.7)	11 (16.9)	16 (6.24)	
Religion				
Buddha	125 (96.2)	63 (96.9)	62 (95.4)	1
Islamic	3 (2.3)	2 (3.1)	1 (1.5)	
Christ	2 (1.5)	-	2 (3.1)	
Education level				
Primary education	93 (71.5)	49 (75.4)	44 (67.7)	1
Secondary education	27 (20.8)	16 (24.6)	11 (16.9)	
Diploma /Bachelor Degree	10 (7.7)	-	10 (15.4)	
Channels received news about cervical cancer				
Health volunteers	88 (34.2)	45 (37.2)	43 (34.4)	0.88
Health care teams	74 (28.8)	38 (31.4)	36 (29.6)	
Television/radio/ close person	66 (26.7)	31 (25.6)	35 (28.0)	
Leaflet	17 (2.3)	7 (5.8)	10 (8.0)	
Medical history of Vaginitis, Leukorrhea, or post intercourse bleeding				
No	109 (83.8)	53 (81.5)	56 (85.2)	0.12
Yes	21 (16.2)	12 (18.5)	9 (13.8)	
Reasons behind not receiving cervical cancer screenings				
A lack time for screening	63 (29.8)	32 (29.4)	31 (30.4)	0.13
Shy health care provider	59 (28.0)	33 (30.3)	26 (25.5)	
Fear of abnormality detection	35 (16.7)	15 (13.8)	20 (19.6)	
Fear of pain	29 (13.7)	13 (11.8)	16 (15.7)	
Lack of knowledge	25 (11.8)	16 (14.7)	9 (8.8)	

p > 0.05 (Independent t-test, Chi-square, and Fisher’s exact test)

**Table 2:** Comparison of Mean Scores and Level of Self-Efficacy Pre-Intervention between the Experimental and the Control Groups Both in Each Dimension and Overall (n = 130)

Self-efficacy	Experimental		Control		*P*-value
	Mean (SD)	Level	Mean (SD)	Level	
1. Strength Dimension	2.20 (.53)	Low	2.27 (.49)	Low	0.45
2. Generality Dimension	2.10 (.51)	Low	2.20 (.45)	Low	0.22
3. Magnitude Dimension	2.23 (.56)	Low	2.18 (.53)	Low	0.65
Total	2.18 (.40)	Low	2.22 (.39)	Low	0.54

**Table 3:** Comparison of Mean Scores and Level of Self-Efficacy Post-Intervention between the Experimental and the Control Groups Both in Each Dimension and Overall (n = 130)

Self-efficacy	Experimental		Control		*P*-value
	Mean (SD)	Level	Mean (SD)	Level	
1. Strength Dimension	3.92 (.61)	High	2.34 (.50)	Moderate	0.001***
2. Generality Dimension	4.23 (.47)	High	2.29 (.56)	Low	0.001***
3. Magnitude Dimension	4.21 (.46)	High	2.20 (.45)	Low	0.001***
Total	4.11 (.44)	High	2.28 (.42)	Low	0.001***

***p < 0.001 (Independent t-test)

**Table 4:** Compare the Number of Screening among Women after Using the Self-Efficacy Enhancement Program

Cervical cancer screening	Week 1	Week 2	Week 3	Week 4	Total
	Number (%)	Number (%)	(%) Number	Number (%)	Number (%)
Experimental	61 (93.9)	3 (4.6)	-	-	64 (98.5)
Control	8 (12.3)	-	-	-	8 (12.3)

**Table 5:** Comparison of Cervical Screening Rates of the Sample Groups after the Self-Efficacy Enhancement Program (n = 130)

Cervical cancer screening	Number (%)		*P*-value
	Come to screening	Not come for screening	
Experimental	64 (98.5)	1 (1.5)	0.001***
Control	8 (12.3)	57 (87.7)	

*P*< 0.001 (Chi- square test)***

## Discussion

The outcome of the self-efficacy enhancement program among women, it was found that women’s mastery experiences were on increasing direct learning experience, providing knowledge about cervical cancer. Women were knowledgeable and right attitude towards cervical cancer screening (Chosamata et al., 2015). Providing education was by the group, and building understanding about the disease by encouraging participation in expressing opinions, causing women to have higher perceived self-efficacy (Staples et al., 2018). Likewise, Kim et al., (2018). They investigated regarding decisions about perceived self-efficacy as a mediator between service providers for cervical cancer screening. 

The women were learning for modeling about to screen for cervical cancer by sharing their experiences and involve women in explaining their problems. The modeling spoke to convince and encourage women to learn from people who were role models, until imitation behaviors will help women overcome their obstacles (Zolfaghari et al., 2018). The study about nursing roles in applying the self-efficacy theory in the elderly found that successfully used of the models allows the patient to see the action of the models (Champatas et al., 2018). Likewise, Prombutr et al., (2019). Investigated the effects of the perceived self-efficacy promotion program on knowledge and perceived self-efficacy of health volunteers, showed that stimulating positive emotions, creating a positive attitude and encouragement were motivation, resulting in higher perceived self-efficacy. Women models can lead to successful expectations, and using the process seriously and continuously will result in women being able to make cervical cancer screening decisions (Sidabutar et al., 2018). Besides, the study of using a confident model to persuade women, reducing fear, and embarrassment for screening for cervical cancer (Kamon et al., 2016). It was causing more women to come for cervical cancer screening.

The results of the self-efficacy enhancement program showed that women came to higher of cervical cancer screening, after used self-efficacy enhancement program, the experimental group came to cervical cancer screening 64 people (98.5%). According to the standard set by the Ministry of Public Health, no less than 80 percent of women required to receive cervical cancer screening (Ministry of Public Health, Strategy and Planning Division, 2017). The control group came to cervical cancer screening 8 people (12.3%). The experimental group came to cervical cancer screening more women in the control group 56 people (86.2%). 

Comparison, the cervical cancer screening in the experimental group and the control group, had differences significantly (p < 0.001), showed that the self-efficacy enhancement program could help develop women to be confident and make a decision to perform cervical cancer screening. Likewise, Zolfaghari et al. (2018), similarly revealed in their study that after the program, 14 women in the experimental group came to receive the screening (20.9%), compared to only seven women from the control group, which indicated statistical significance. Comparison with the criteria of the Ministry of Public Health, it was found that the screening criteria had not been passed yet, because of a lack of preparation for the women health volunteers and models in monitoring and persuading women to screen for cervical cancer. 

The result of this research showed that women with high perceived self-efficacy could decide to decide for cervical cancer screening successfully by themselves. They were caused by women who have the intention to take care of their health and have confidence in themselves, including awareness of correct cervical cancer prevention (Sidabutar et al., 2018). Therefore, they should promote the capacity of self for women, help women get screened for early cervical cancer, can reduce the incidence rate and mortality rate of cervical cancer.

In conclusion, the self-efficacy enhancement program was for women, consider the area, the background, focus on giving women the confidence to overcome the obstacles to cervical cancer screening (Chosamata et al., 2015). According to the program implementation plan make an appointment, attended the activity to 2 times, to create awareness and confidence for women to see the importance of screening for cervical cancer by providing knowledge, successful exchange of experiences, participation and comment, persuading from modeling to an exposure to convince women. Self-efficacy enhancement program development can be applied to the context in the area because it used only 2 weeks to implement the program. The program takes less time, significantly reducing the workload of public health personnel, helps to make the effectiveness of cervical cancer screening operations successful following the criteria of the Ministry of Public Health.

## References

[B1] Alnafisah R, Alsuhaibani R, Alharbi M, Alsohaibani A, Ismai A (2019). Saudi woman’s knowledge and attitude toward cervical cancer screening, treatment, and prevention: A cross-sectional study in Qassim Region (2018-2019). Asian Pac J Cancer Prev.

[B2] Bandura A (1977). Self-efficacy: Toward unifying theory of behavioral change. Psychol Rev.

[B3] Bandura A (1997). Self-efficacy: The exercise of control.

[B4] Champatas N, Jamjang L, Waimuanchai P (2018). The nurse’s role for apply self- efficacy theory in the elderly diabetic. J Huachiew Chalermprakiet Univ.

[B5] Chosamata M, Hong S, Tiraphat S (2015). Determinants of cervical cancer screening utilization among women aged 30-45 years in Blantyre district, Malawi. J Public Health Dev.

[B6] Gatumo M, Gacheri S, Sayed A, Scheibe A (2019). Women’s knowledge and attitudes related to cervical cancer and cervical cancer screening in Isiolo and Tharaka Nithi countries, Kenya: A cross-sectional study. BMC Cancer.

[B7] Hair J, Black W, Babin B, Anderson R (2010). Multivariate data analysis (7th ed).

[B8] Izadirad H, Niknami S, Zareban I, Hidarnia A (2017). Effects of social support and self-efficacy on maternal prenatal cares among the first-time pregnant women, Iranshahr, Iran. JFamily Reprod Health.

[B9] Kamon R, Kangpanit T, Kangpanit M, Benjakul S (2016). The application of health belief model to promote cervical cancer screening among women aged 30-60 years performed by women health volunteer. J Nurs Public Health Edu.

[B10] Kelly H, Mayaud P, Weiss H (2017). The epidemiology of human papillomavirus (HPV) infection and epigenetic factors associated with the development of cervical cancer precursor lesions in women living with HIV in Africa.

[B11] Kim K, Xue QL, Walton-Moss B, Nolan MT, Han HR (2018). Decisional balance and self-efficacy mediates the association among provider advice, health literacy and cervical cancer screening. Eur J Oncol Nurs.

[B12] Maneechot P, Songwatthana P, Kritcharoen K (2011). The effects of motivated teaching program on disease perception and cervical cancer screening rate among rural Thai women. Srinagarind Med J.

[B13] Maniar K, Wei J (2017). Pathology of cervical carcinoma. The Global Library of Women’s Medicine.

[B14] Ministry of Public Health (2019). Cervical cancer rate in age 30-60 years.

[B16] Ministry of Public Health, Strategy and Planning Division (2017). Public health statistics.

[B17] Prombutr P, Sookpool A, Phinyo K, Phinyo P (2015). The effect of self-efficacy program toward knowledge and self-efficacy of village Health volunteers in diabetic and hypertension patient care in community in Northeast Province. Songklanagarind J Nurs.

[B18] Rimande-Joel R, Ekenedo G (2019). Knowledge, Belief and practice of cervical cancer screening and prevention among women of Taraba, North-East Nigeria. Asian Pac J Cancer Prev.

[B19] Sakaew R, Thanasai J, Wannaprapan B (2017). Effect of a health behavior promoting program for cervical cancer screening among 30 to 60 years old women at Nonglek Health Center, Sikhoraphum District, Surin Province. The Journal of Baromarajonani College of Nursing, Nakhonratchasima.

[B20] Sidabutar S, Suwani T, Martini S, Hargono R (2018). Factors influencing decision conduct early detection of cervical cancer. Health Notions.

[B21] Staples JN, Wong MS, Rimel BJ (2018). An educational intervention to improve human papilloma virus (HPV) and cervical cancer knowledge among African American college students. Gynecol Oncol.

[B22] Suksawat S, Junprasert S, Sananreangsak S, Tachasuksri T, Aungkaprasatchai W (2017). A development of health promotion curriculum in cervical cancer prevention of nursing learning center. J Nurs Sci Chulalongkorn Univ.

[B23] Suratthani Provincial Health Office (2018). HDC online Suratthani.

[B24] World Health Organization (2018). WHO Director-General calls for all countries to take action to help end the suffering caused by cervical cancer.

[B25] Zolfaghari Z, Rezaee N, Shakiba M, Navidian A (2018). Motivational interviewing-based training vs traditional training on the uptake of cervical screening: A quasi-experimental study. Public Health.

